# Pharmacometabolomics of sulfonylureas in patients with type 2 diabetes: a cross-sectional study

**DOI:** 10.3389/jpps.2024.13305

**Published:** 2024-09-17

**Authors:** Khaled Naja, Najeha Anwardeen, Sara S. Bashraheel, Mohamed A. Elrayess

**Affiliations:** ^1^ Biomedical Research Center, Qatar University, Doha, Qatar; ^2^ College of Medicine, QU Health, Qatar University, Doha, Qatar

**Keywords:** sulfonylureas, type 2 diabetes, metabolic signatures, precision medicine, metabolomics

## Abstract

**Background:**

Sulfonylureas have been a longstanding pharmacotherapy in the management of type 2 diabetes, with potential benefits beyond glycemic control. Although sulfonylureas are effective, interindividual variability exists in drug response. Pharmacometabolomics is a potent method for elucidating variations in individual drug response. Identifying unique metabolites associated with treatment response can improve our ability to predict outcomes and optimize treatment strategies for individual patients. Our objective is to identify metabolic signatures associated with good and poor response to sulfonylureas, which could enhance our capability to anticipate treatment outcome.

**Methods:**

In this cross-sectional study, clinical and metabolomics data for 137 patients with type 2 diabetes who are taking sulfonylurea as a monotherapy or a combination therapy were obtained from Qatar Biobank. Patients were empirically categorized according to their glycosylated hemoglobin levels into poor and good responders to sulfonylureas. To examine variations in metabolic signatures between the two distinct groups, we have employed orthogonal partial least squares discriminant analysis and linear models while correcting for demographic confounders and metformin usage.

**Results:**

Good responders showed increased levels of acylcholines, gamma glutamyl amino acids, sphingomyelins, methionine, and a novel metabolite 6-bromotryptophan. Conversely, poor responders showed increased levels of metabolites of glucose metabolism and branched chain amino acid metabolites.

**Conclusion:**

The results of this study have the potential to empower our knowledge of variability in patient response to sulfonylureas, and carry significant implications for advancing precision medicine in type 2 diabetes management.

## Introduction

Sulfonylureas, the oldest oral antidiabetic agents, remain one of the most commonly prescribed drugs for the treatment of type 2 diabetes (T2D), along with metformin [1]. Sulfonylureas are recommended as an add-on to metformin therapy in most international guidelines, and they can be used in combination with all classes of oral antidiabetics except glinides [2]. The use of sulfonylureas as a monotherapy has decreased over time, and it is mainly endorsed for patients who cannot use metformin [3]. However, sulfonylureas have been found to have pleiotropic effects beyond their use in diabetes management which opens up the possibility of repurposing these drugs [4]. Sulfonylureas can also be used as third-line agents for the management of uncontrolled diabetes with dual combination therapy [5].

Sulfonylureas are insulin secretagogues that function through stimulating insulin secretion from the pancreatic beta-cell (β-cell). Specifically, sulfonylureas bind to their specific receptors on β-cell and inhibit the ATP-sensitive potassium channels (K_ATP_) on the membrane of β-cell, causing potassium efflux to decrease and the β-cell membrane to depolarize. This depolarization leads to calcium channels opening which results in calcium influx and increased intracellular calcium leading to a contraction of the filaments of actomyosin responsible for the exocytosis of insulin, and ultimately insulin secretion [6]. Sulfonylureas are usually well tolerated, however, they may cause side effects such as hypoglycemia and weight gain [7].

It is noteworthy that a significant proportion of patients do not respond optimally to sulfonylurea treatment [8]. Pharmacogenomic studies have revealed the influence of genetic factors on individual responses to sulfonylureas. Genetic variants in genes like KCNQ1, KCNJ11, ABCC8, CYP2C9, and TCF7L2 can influence the therapeutic response and adverse effects of sulfonylureas [9, 10]. However, research has shown conflicting results across various studies, highlighting the complexity of understanding how genetic factors contribute to sulfonylurea responses [9]. Metabolomics is a rapidly evolving field with the potential to significantly impact the future of precision medicine. It offers valuable insights into individualized treatment strategies, enhances our understanding of diseases, and enables the assessment of therapeutic efficacy [11].

The objective of this study is to identify specific metabolic signatures that are correlated with good and poor responses to sulfonylureas. This is being pursued through a retrospective cross-sectional study that is focusing on identifying blood metabolites linked to each category of sulfonylureas response. Comprehending the role of these metabolites can help tailor sulfonylurea therapy, thereby enhancing treatment effectiveness, and advancing the progressive shift toward precision medicine in diabetes management.

## Materials and methods

### Data source and study participants

This research gathered information from individuals recruited at Qatar Biobank (QBB). The QBB database encompasses a comprehensive profile of Qatari citizens or long-term residents (≥15 years residing in Qatar), aged 18 years and above, within the State of Qatar. Detailed baseline socio-demographic, clinical and behavioral phenotypic data, as well a wide range of biochemical parameters were assessed at the central laboratory of Hamad Medical Corporation (HMC), which is accredited by the College of American Pathologists. QBB data also included questionnaires related to their history of diabetes, medication usage, and metabolomics data for more than 1,000 metabolites. The research was approved by the Institutional Review Boards of the Qatar Biobank. All participants provided informed consent. Among the participants, a total of 137 patients with Type 2 Diabetes who were taking sulfonylurea in monotherapy or combination therapy and had available metabolic data were selected and included in this study. Patients with incomplete or inconsistent medication records were excluded. Among them, 41 patients were on sulfonylurea monotherapy (Gliclazide), 63 on dual therapy (59 combined with metformin, 2 combined with sitagliptin, and 2 combined with pioglitazone), and 33 on triple therapy (28 combined with both metformin and sitagliptin, and 5 combined with both metformin and pioglitazone). Daily doses of gliclazide modified release range from 30 mg to 120 mg, and for gliclazide immediate release from 80 mg to 320 mg. Patients were empirically dichotomized according to their HbA1C levels, which is the most widely used measure of glycemic control [12] into poor responders (HbA1C ≥ 7), and good responders (HbA1C < 7) in accordance with the American Diabetes Association guidelines and previous studies [13, 14].

### Metabolomics

All participant serum samples were subjected to untargeted metabolomics using established protocols by Metabolon [15]. Metabolites measurement was performed using a Thermo Scientific Q-Exactive high resolution/accurate mass spectrometer (Thermo Fisher Scientific, Inc., Waltham, MA, United States) interfaced with a heated electrospray ionization (HESI-II) source and Orbitrap mass analyzer operated at 35,000 mass resolution along with Waters ACQUITY ultra-performance liquid chromatography (UPLC) (Waters Corporation, Milford, MA, United States). A thorough explanation of the process has already been provided [15]. Hits were matched with pre-existing library entries of over 3,300 pure standard chemicals to identify the compounds. Compounds were divided into several groups according to their sources. Internal standards and quality checks have been previously published [16]. In short, to adjust for discrepancies in sample preparation and instrument performance, a combination of stable isotope-labeled chemicals was utilized as internal standards. The stability and repeatability of the procedure were tracked over time using quality control samples. To reduce variability and guarantee the integrity of the samples, a systematic methodology was employed for pre-analytical sample management including sample collection, storage, and preparation.

### Statistical analysis

The metabolomics data were inverse rank normalized. SIMCA^®^ and R software were used to conduct multivariate analysis and linear models respectively. Linear regression analysis was performed to for each metabolite as y and good/poor responder grouping variable as x. The model also contained age, gender, body mass index (BMI), metformin usage (yes/no) and principal components 1 and 2 as confounders. To ensure the robustness of our findings, we have meticulously adjusted our analysis to account for any confounding effects of concurrent metformin treatment. This adjustment is crucial as it isolates the specific metabolic effects attributable to sulfonylureas, thereby providing a clearer understanding of the metabolomic variations associated with their response. The nominal *p*-values were adjusted using the multiple testing correction method (False Discovery Rate, FDR). Statistical significance was defined as FDR < 0.05. Functional enrichment analysis was performed on all nominally significant metabolites using Wilcoxon sum of ranks test and was followed by *p*-value adjustment using FDR method. Additional details of the statistical analysis has previously been provided [17].

## Results

### General characteristics of participants

One hundred and thirty-seven patients with T2D were categorized into “poor responders” (n = 85) and “good responders” (n = 52) based on their HbA1C levels. Table 1 reveals significantly higher levels of fasting blood glucose, HbA1C, homeostatic model assessment of insulin resistance (HOMA-IR), and gamma-glutamyl transferase (GGT) in the poor response group when compared to the good response one; whereas the good response group reveals significantly higher levels of C-peptide and chloride.

**TABLE 1:** General characteristics of participants.

Test	Variable	Poor responders (N = 85)	Good responders (N = 52)	*P*-value
General characteristics	Gender (M/F)	40/45	32/20	0.141
	Age	53 (46–58)	50.5 (40.75–58.25)	0.304
	Metformin use (Y/N)	66/19	26/26	0.002
	BMI (kg/m^2^)	30.41 (26.56–34.63)	29.69 (27.61–34.03)	0.755
	Systolic blood pressure (mmHg)	128.06 (15.3)	121.02 (15.89)	0.018
	Diastolic blood pressure (mmHg)	76.59 (10.84)	73.94 (10.94)	0.088
Blood sugar	Fasting blood glucose (mmol/L)	11.1 (9.1–15.3)	6.75 (5.39–8.22)	<0.001*
	HbA_1C_ (%)	9 (8.2–10.4)	6.1 (5.9–6.4)	<0.001*
	C-peptide (ng/mL)	2.7 (1.88–3.93)	3.48 (2.28–5.86)	0.009*
	HOMA-IR	10.06 (5.43–18.24)	4.04 (2.4–12.26)	0.001*
	Insulin (uU/mL)	18.5 (12–36.9)	13 (8.97–38.42)	0.388
Lipid profile	Total cholesterol (mmol/L)	4.67 (4.2–5.21)	5 (4.1–5.53)	0.204
	HDL-cholesterol (mmol/L)	1.1 (0.96–1.37)	1.1 (0.92–1.41)	0.645
	LDL-cholesterol (mmol/L)	2.75 (2.02–3.18)	3 (2.07–3.5)	0.245
	Triglyceride (mmol/L)	1.55 (1.2–2.2)	1.96 (1.1–2.4)	0.688
Kidney function	Creatinine (µmol/L)	63 (51–76)	69.5 (54.75–85.25)	0.038
	Chloride	99 (97–100)	101 (100–102)	<0.001*
	Urea (mmol/L)	4.5 (3.7–5.1)	4.65 (3.7–5.2)	0.447
	Bicarbonate (mmol/L)	26.66 (2.02)	26.69 (2.41)	0.927
	Total protein (g/L)	72.54 (3.81)	71.87 (3.9)	0.364
Heart function	NT-proBNP	26.15 (15.35–44.5)	27.05 (13.57–39.33)	0.842
Liver function	Albumin (g/L)	44 (2.83)	44.33 (2.5)	0.519
	ALT (U/L)	24 (19–32)	20.5 (16–28)	0.067
	AST (U/L)	19 (15–23)	17 (14–20.25)	0.152
	GGT (U/L)	32 (23.5–73.5)	16.5 (12.75–20)	0.001*
Hormones	TSH (mIU/L)	1.37 (0.94–2.08)	1.33 (0.9–1.86)	0.902
	Free thyroxine (pmol/L)	13.6 (12.6–14.34)	12.95 (11.84–13.67)	0.015
	Free triiodothyronine (pmol/L)	4.2 (3.9–4.56)	4.35 (4.02–4.53)	0.411

Data are presented as mean (SD), median (IQR) and number for parametric, non-parametric, and nominal variables respectively. The difference between mean/median was evaluated using independent *t*-test/Mann-Whitney *U* test as appropriate. Chi-square test was used for nominal variable. *Bolded *P*-value indicates significant difference between the two studied groups. Abbreviations: BMI, body mass index; HbA_1C_, glycosylated hemoglobin; HOMA-IR, homeostatic model assessment of insulin resistance; HDL, high-density lipoprotein; LDL, low-density lipoprotein; ALT, alanine transaminase; AST, aspartate aminotransferase; GGT, gamma-glutamyl transferase; TSH, thyroid stimulating hormone; NT-proBNP, N-terminal pro b-type natriuretic peptide.

### Multivariate analysis of metabolites differentiating poor and good sulfonylurea responders

**FIGURE 1:**
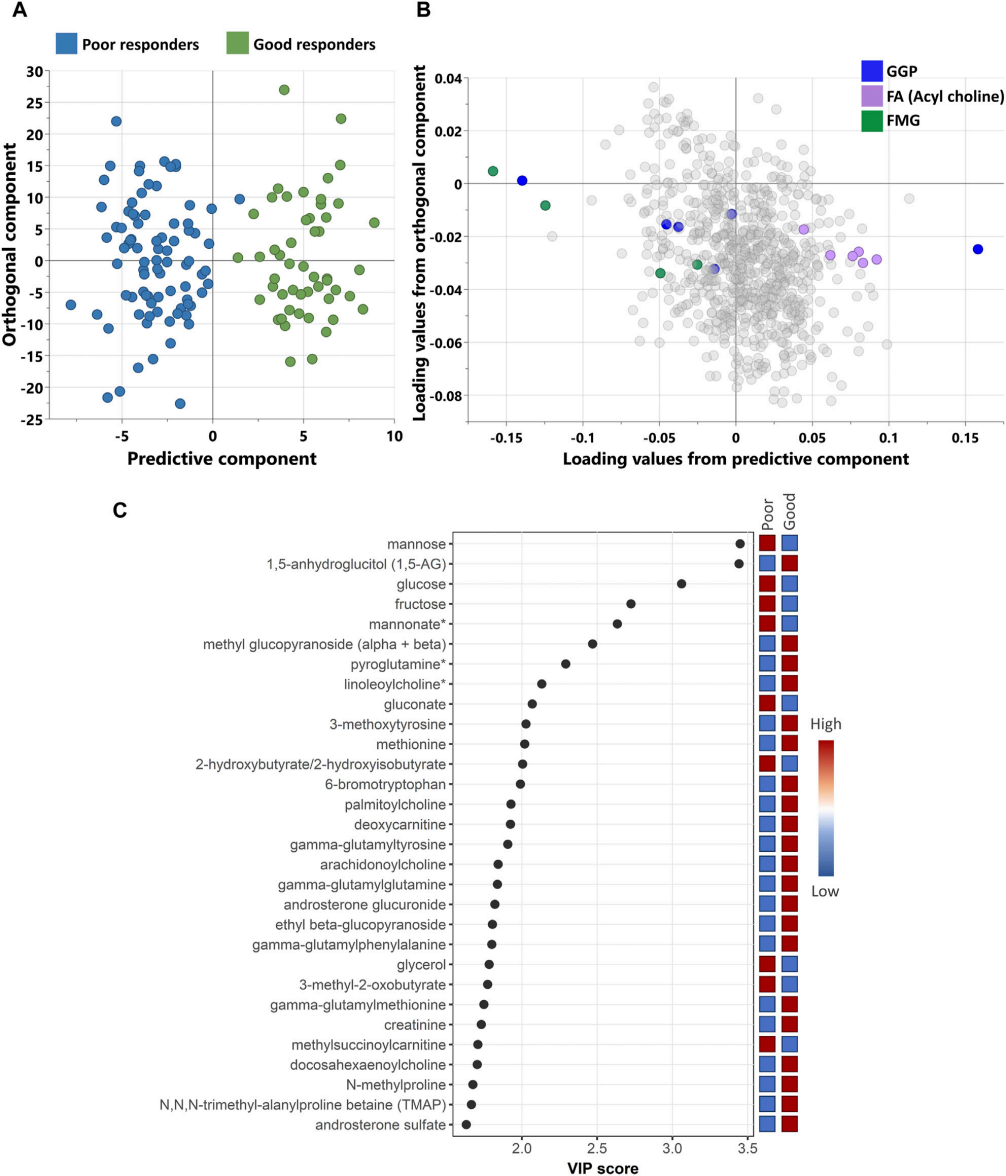
**(A)** scores and **(B)** loading plots from OPLS-DA between poor and good response. The model shows a good separation between the study groups with R2Y = 85.2% and Q2 = 49.1%. **(C)** A scatterplot of VIP score of the top 30 metabolites showing which metabolites are most useful in distinguishing between responders’ groups. (*) indicates a compound that has not been officially confirmed based on a standard but that Metabolon is confident in its identity. GGP, Glycolysis, Gluconeogenesis, and Pyruvate metabolism; FA, Fatty Acid metabolism; FMG, Fructose, Mannose and Galactose metabolism.

**FIGURE 2:**
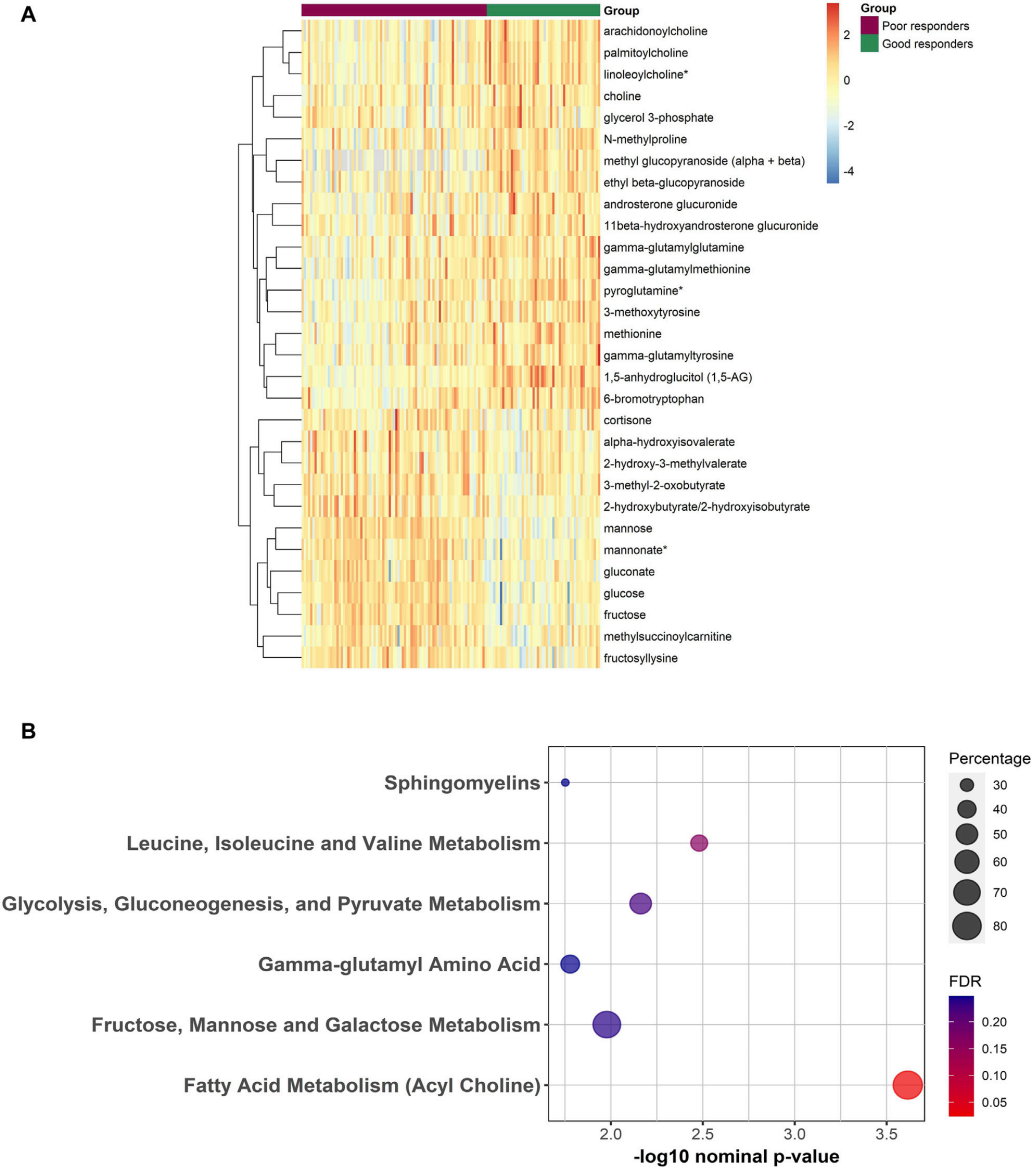
**(A)** Heatmap representing the abundance of FDR significant metabolites between poor and good responders from the linear regression analysis. Red/Blue correspond to more/less abundance, respectively. **(B)** Bubble plot depicting the enriched pathways from the functional enrichment analysis using Wilcoxon sum of ranks test. Percentage reflects the number of significantly different metabolites divided by total number of metabolites from that pathway.

Non-targeted metabolomics analysis was performed to investigate the metabolic signatures of 137 patients with Type 2 Diabetes (T2D) who were taking sulfonylureas. Orthogonal partial least squares discriminant analysis (OPLS-DA) was used to identify the best distinguishing components between poor and good responders as shown in Figure 1. OPLS-DA showed one predictive and two orthogonal components, with the discriminatory component accounting for 85.2% of the variance between poor and good responders. Figure 2C shows the list of metabolites with VIP > 1.5.

### Univariate analysis of metabolites differentiating poor and good sulfonylurea responders

Linear model analysis revealed a number of FDR (≤0.05) significant changes between the two studied groups (Table 2). This includes an increase in 1,5-anhydroglucitol, 6-bromotryptophan, 3-methoxytyrosine, methionine, pyroglutamine, ethyl and methyl glucopyranoside, gamma-glutamyl amino acids and acylcholines in the good responders group. Whereas an increase in glucose, mannose, fructose, mannonate, gluconate, fructosyl-lysine, and many branched chain amino acid metabolites was shown in the poor responders group. Supplementary Table S1 contains raw data values with standard deviations. The analysis conducted separately for males and females produced similar results except for 6-bromotryptophan which differentiate good responders from poor responders in males only (Supplementary Table S2).

**TABLE 2:** Top metabolites differentiating good responders from poor responders.

Metabolites	Sub-pathway	Superpathway	Estimate	SE	*p*-value	FDR
1,5-anhydroglucitol (1,5-AG)	Glycolysis, Gluconeogenesis, and Pyruvate Metabolism	Carbohydrate	1.408	0.128	3.29 × 10^−20^	2.76 × 10^−17^
Mannose	Fructose, Mannose and Galactose Metabolism	Carbohydrate	−1.276	0.147	1.41 × 10^−14^	5.90 × 10^−12^
Glucose	Glycolysis, Gluconeogenesis, and Pyruvate Metabolism	Carbohydrate	−1.229	0.158	2.07 × 10^−12^	5.80 × 10^−10^
Fructose	Fructose, Mannose and Galactose Metabolism	Carbohydrate	−1.024	0.165	6.24 × 10^−9^	1.31E-06
Pyroglutamine*	Glutamate Metabolism	Amino Acid	0.831	0.153	2.77 × 10^−7^	4.65 × 10^−5^
Methyl glucopyranoside (alpha + beta)	Food Component/Plant	Xenobiotics	1.163	0.218	6.14 × 10^−7^	8.59 × 10^−5^
Mannonate*	Food Component/Plant	Xenobiotics	−0.793	0.152	7.48 × 10^−7^	8.97 × 10^−5^
6-bromotryptophan	Tryptophan Metabolism	Amino Acid	0.879	0.184	4.92 × 10^−6^	5.17 × 10^−4^
Palmitoylcholine	Acyl Choline	Lipid	0.884	0.187	5.69 × 10^−6^	5.31 × 10^−4^
Linoleoylcholine*	Acyl Choline	Lipid	0.862	0.184	7.07 × 10^−6^	5.94 × 10^−4^
Gluconate	Food Component/Plant	Xenobiotics	−0.695	0.156	1.83 × 10^−5^	1.39 × 10^−3^
Methylsuccinoylcarnitine	Leucine, Isoleucine and Valine Metabolism	Amino Acid	−0.790	0.182	2.85 × 10^−5^	2.00 × 10^−3^
3-Methoxytyrosine	Tyrosine Metabolism	Amino Acid	0.814	0.189	3.34 × 10^−5^	2.16 × 10^−3^
Ethyl beta-glucopyranoside	Food Component/Plant	Xenobiotics	0.787	0.184	3.76 × 10^−5^	2.26 × 10^−3^
3-methyl-2-oxobutyrate	Leucine, Isoleucine and Valine Metabolism	Amino Acid	−0.749	0.180	5.58 × 10^−5^	2.83 × 10^−3^
Arachidonoylcholine	Acyl Choline	Lipid	0.775	0.186	5.78 × 10^−5^	2.83 × 10^−3^
Choline	Phospholipid Metabolism	Lipid	0.729	0.176	5.84 × 10^−5^	2.83 × 10^−3^
Methionine	Methionine, Cysteine, SAM and Taurine Metabolism	Amino Acid	0.693	0.167	6.07 × 10^−5^	2.83 × 10^−3^
Gamma-glutamylglutamine	Gamma-glutamyl Amino Acid	Peptide	0.784	0.190	6.40 × 10^−5^	2.83 × 10^−3^
Glycerol 3-phosphate	Glycerolipid Metabolism	Lipid	0.755	0.193	1.52 × 10^−4^	6.38 × 10^−3^
3-hydroxybutyrate/2-hydroxyisobutyrate	Glutathione Metabolism	Amino Acid	−0.714	0.184	1.62 × 10^−4^	6.49 × 10^−3^
Fructosyl-lysine	Lysine Metabolism	Amino Acid	−0.678	0.182	2.94 × 10^−4^	1.09 × 10^−2^
N-methyl proline	Urea cycle; Arginine and Proline Metabolism	Amino Acid	0.767	0.206	2.97 × 10^−4^	1.09 × 10^−2^
Gamma-glutamyl tyrosine	Gamma-glutamyl Amino Acid	Peptide	0.588	0.170	7.27 × 10^−4^	2.54 × 10^−2^
Androsterone glucuronide	Androgenic Steroids	Lipid	0.598	0.174	7.89 × 10^−4^	2.65 × 10^−2^
11beta-hydroxyandrosterone glucuronide	Androgenic Steroids	Lipid	0.555	0.168	1.24 × 10^−3^	3.93 × 10^−2^
Alpha-hydroxyisovalerate	Leucine, Isoleucine and Valine Metabolism	Amino Acid	−0.588	0.179	1.26 × 10^−3^	3.93 × 10^−2^
2-hydroxy-3-methylvalerate	Leucine, Isoleucine and Valine Metabolism	Amino Acid	−0.573	0.175	1.32 × 10^−3^	3.95 × 10^−2^
Gamma-glutamyl methionine	Gamma-glutamyl Amino Acid	Peptide	0.592	0.184	1.59 × 10^−3^	4.60 × 10^−2^

### Functional enrichment analysis

Results of functional enrichment analysis (Table 3) indicated significant differences in pathways of acylcholines, gamma-glutamyl amino acids, sphingomyelins, branched chain amino acid metabolites, fructose, mannose and galactose metabolism, and in glycolysis, gluconeogenesis, and pyruvate metabolism. Heatmap showing the top metabolites is also shown in Figure 2.

**TABLE 3:** Results from functional enrichment analysis based on metabolite ranks by *p*-value using the Fisher’s exact test.

Enriched pathways	*p*-value	FDR
Acyl Choline	0.000	0.024
Leucine, Isoleucine and Valine Metabolism	0.003	0.162
Glycolysis, Gluconeogenesis, and Pyruvate Metabolism	0.007	0.225
Fructose, Mannose and Galactose Metabolism	0.011	0.239
Gamma-glutamyl Amino Acid	0.017	0.248
Sphingomyelins	0.018	0.248

## Discussion

Sulfonylureas have been a longstanding option in the treatment armamentarium of T2D. Sulfonylureas exert their therapeutic effects on pancreatic beta cells by promoting calcium influx into these cells and ultimately increasing insulin secretion. Pharmacogenomics may offer some insight into the variable responses to sulfonylurea which lead to better or worse glycemic control. However, the current estimate suggests that genetics contributes to approximately 20%–40% of the individual variation in drug responses [18]. Pharmacometabolomics is sensitive to both genetic and environmental influences. Therefore, it is a potent method for elucidating variations in individual drug response. In this cross-sectional study, we have identified metabolic signatures associated with poor and good responders to sulfonylurea in a set of 137 samples from the Qatar Biobank. To the best of our knowledge, this study represents the first comparative analysis of the metabolic profiles between poor and good responders to sulfonylurea therapy.

Expectedly, good responders showed significant higher levels of 1,5-anhydroglucitol (1,5-AG), while poor responders showed higher levels of glucose, mannose, fructose, and fructosyl-lysine. These metabolites reflect the level of glycemia, and the regulated/dysregulated glucose metabolism in each response category [19]. Good responders exhibited significantly elevated levels of C-peptide, indicative of the effective functioning of pancreatic β-cells. Higher C-peptide levels have been associated with a better response to certain antidiabetics like metformin, sulfonylureas, and thiazolidinediones [20]. Poor responders showed higher levels of GGT; the increased concentrations of this enzyme has been associated with a poor glycemic control [21].

Remarkably, the good response group was associated with a reduction in branched-chain ketoacids (BCKA), and branched-chain alpha-hydroxy acids (BCHA) which are intermediates formed during the catabolism of branched chain amino acid (BCAA) [22]. These intermediates were associated with metabolic disorders [23], and recently have been shown to contribute to pathologic cardiac hypertrophy [24]. Given that the cardiovascular safety of sulfonylureas is still a topic of debate [25], it is important in future studies to consider the potential role of BCKA and BCHA in this context. BCKAs have also been shown to chronically suppress the serine/threonine kinase 2 (akt2) [26]. Strikingly, the activity of akt2 is essential for the proper functioning, survival, and adaptive growth of β-cells [27, 28]. Moreover, Sirtuin 4 (SIRT4) which plays a crucial role in the regulation of BCAA catabolism, is expressed in islets of Langerhans, and has been shown to control insulin secretion [29, 30]. This suggests a potential link between BCKA and BCHA with insulin secretion in the context of SU treatment, and opens up exciting new avenues for investigating their potential role in the health of β-cells.

Our emerging results showed an increase of many acylcholines in the sulfonylurea responsive group, supported by an FDR significant enriched metabolic pathway. Data on the biological activity of acylcholines remain very limited. Recently, they are discovered as endogenous modulators of the acetylcholine signaling system through the inhibition of the enzyme acetylcholinesterase [31]. Interestingly, sulfonylureas have been shown to inhibit acetylcholinesterase activity [4]. Acetylcholine is crucial for pancreatic beta cell function. It stimulates insulin secretion by acting on M3 muscarinic receptors on beta-cells [32], and thus increasing the concentration of cytoplasmic free calcium [33]. Considering that the M3 muscarinic receptors serve as promising targets for innovative antidiabetic medications, acylcholines could present novel opportunities in this context.

Interestingly, good responders to sulfonylureas exhibited higher levels of many gamma-glutamyl amino acids. These metabolites are dipeptides consisting of a C-terminal amino acid having a gamma-glutamyl residue attached at the N alpha-position. In recent years, gamma-glutamyl amino acids have caught researchers’ attention due to their ability to allosterically activate the calcium-sensing receptor (CaSR) [34]. CaSR is expressed in β-cells, and plays an important role in regulating its function by influencing expression and function of potassium and voltage-dependent calcium channels, and by controlling cell adhesion, coupling, and communication [35–37]. Further research is required to clarify the role of gamma-glutamyl amino acids in glucose homeostasis.

Our emerging results also showed that sphingomyelins metabolic pathway was nominally enriched in the responsive group. In fact, Sphingomyelins play a significant role in the function of pancreatic beta cells. They are involved in regulating beta-cell excitability and insulin exocytosis [38]. Griess et al. demonstrated that a β-cell-specific ablation of ceramide synthase 2, the enzyme necessary for generation of very-long-chain sphingolipids, selectively reduces insulin content and impairs insulin secretion [39]. Khan et al. reported that a downregulated sphingolipid impairs pancreatic β cell function [40]. Several phospholipids, particularly sphingomyelins, were shown to be significantly lower in the serum of children who later progress to T1DM [41]. Additionally, a beta-cell-specific antibody has been found to target unique sphingomyelin patches on the surface of live cells [42]. Interestingly, a deficiency in the enzyme sphingomyelin synthase 1 leads to a reduction in KCNQ1 expression, and the phospho-head group in sphingomyelin is essential for the proper gating of certain voltage-dependent K^+^ channels [43]. Sphingomyelins are also shown to be associated with ATP-binding cassette (ABC) proteins, and ABCA7 deficiency impairs sphingomyelin synthesis [44]. A review stipulated that lower sphingomyelin may be associated with higher risks of coronary heart disease and T2D [30]. We hypothesize that elevated sphingomyelin could indicate an improved β cell function reflecting a good response to sulfonylureas, which might be associated with a lower risk of heart disease.

Univariate analysis showed the association of gut-microbiota metabolites, namely, ethyl glucopyranoside, methyl glucopyranoside, mannonate and gluconate, with the response to sulfonylureas. While no study has yet explored the direct impact of sulfonylureas on the gut microbiota, recent research indicates that sulfonylureas may indeed have some interaction with the gut microbiota [45]. Research also highlights the role of gut microbiota-derived metabolites in influencing β-cell function by playing a crucial role in protecting β-cell and promoting insulin secretion [46]. Interestingly, ethyl and methyl glucopyranoside associated in our study with the good responders to sulfonylureas, were identified to be associated with better microbiome diversity and a healthier status [47]. While a review concluded the lack of an alteration in the gut microbiota by sulfonylureas [48], it is important to note that the interaction between sulfonylureas and gut microbiota may not be bidirectional. Our hypothesis suggests that a dysbiosis in the gut microbiota might be linked to a poor response to sulfonylureas.

Univariate analysis also showed the association of pyroglutamine, methionine, and 3-metoxytyrosine with the good response group. Indeed, pyroglutamine (also called 5-oxoproline) is involved in glutathione metabolism, which plays a role in maintaining redox homeostasis in pancreatic β-cells [49]. Methionine is known to regulate insulin secretion from pancreatic β-cells [50], and methionine accumulation in the pancreas has been reported to represent β-cell function [51]. The metabolite 3-metoxytyrosine is a product in the metabolism of dopamine, the latter has been reported to regulate pancreatic insulin secretion via adrenergic and dopaminergic receptors [52]. However, the data on the function of 3-metoxytyrosine remains very scarce, and much work still needs to be done to elucidate its exact functions and significance.

Strikingly, our results showed a significant association between a novel metabolite, namely, 6-bromotryptophan with the good response group especially in males. 6-bromotryptophan is a metabolite derived from the bromination of tryptophan. While it is believed that 6-bromotryptophan is a microbiome-derived metabolite, its formation and the localization of this reaction are not well understood. It is accepted that 6-bromotryptophan has a strong relationship with fat distribution [53], and recently, it has been associated with lower risk for chronic kidney progression in multiple studies [54]. Taking into consideration that low urinary chloride concentration was associated with a higher risk for CKD progression [55], our results are corroborated with the significant difference (*p* < 0.001) in chloride levels between good and poor responders. The BROMO clinical trial (NCT05971524) is currently underway to investigate the safety, pharmacokinetics, and efficacy of dietary supplement of 6-bromotryptophan in individuals with metabolic syndrome. Interestingly, the same clinical trial claims that 6-bromotryptophan was positively associated with C-peptide and preserved β-cell function. Relatedly, a study on the therapeutic applications of a 6-bromotryptophan-containing conotoxin demonstrated its bioactivity on insulin release in pancreatic β-cell [56]. Indeed, tryptophan is necessary in all KCNQ subunits to confer drug sensitivity [57]. Moreover, the variant rs7903146 in TCF7L2 gene has been associated with the therapeutic response to sulfonylureas. The rs7903146 T-allele conferred a higher risk for sulfonylurea treatment failure by impairing β-cell function [58, 59]. Interestingly, serotonin, synthesized solely from tryptophan, was identified as the top metabolite being increased in carriers of the rs7903146 risk allele [60]. It is important to note that an allosteric modulator of β-cell M3 muscarinic receptors (VU0119498) [61], which stimulates insulin release and improves glucose homeostasis, has a similar structure to 6-bromotryptophan. Taken together with the literature, we present evidence supporting the involvement of tryptophan metabolism in pancreatic β-cell function. The proxy metabolite 6-bromotryptophan may serve as an indicator of a positive response, or even as a candidate for drug development. Nevertheless, further research is essential to clarify the connection between 6-bromotryptophan and its related metabolites, and the response to sulfonylurea treatment.

## Conclusion

This study offers the research community with a wealth of metabolic biomarkers associated with response to sulfonylurea, notably branched chain amino acid metabolites, sphingomyelins, acylcholines, gamma glutamyl amino acids, and the novel metabolite 6-bromotryptophan. The study’s findings support the development of biomarker-driven treatment strategies that can lead to more personalized and effective diabetes management, and we believe these findings can be translated into clinically relevant metabolic signatures, enabling further research into personalized therapeutic approaches.

While the confounding effect of metformin has been corrected in this study, we acknowledge the presence of some limitations. One limitation of this study is its cross‐sectional design, which makes it challenging to determine if individuals with elevated HbA1C levels exhibited a poor response to sulfonylurea, or had more advanced diabetes prior to starting treatment. Consequently, certain metabolites may be linked to the progression or complications of diabetes rather than treatment response. Moreover, the QBB data lacks baseline metabolomics information, making it difficult to compare metabolic profiles before and after treatment. Additionally, the variability in sulfonylurea dosages across participants, and the absence of data regarding the duration of treatment, present another potential limitation. Future research involving longitudinal studies will thus be warranted to validate our identified biomarkers.

## Data Availability

The data analyzed in this study is subject to the following licenses/restrictions: The datasets used and/or analyzed during the current study are available from the corresponding author on reasonable request. Requests to access these datasets should be directed to m.elrayess@qu.edu.qa.
