# Supplementary material for: Pharmacometabolomics of sulfonylureas in patients with type 2 diabetes: a cross-sectional study
Source: J Pharm Pharm Sci. 2024 Sep 17;27:13305. doi: 10.3389/jpps.2024.13305 (PMC11442225; doi:10.3389/jpps.2024.13305)
Supplement: Supplementary file 1 [file DataSheet1.docx]

**Table S1**. Raw data values of the top metabolites differentiating good responders from poor responders.

| Metabolites | Sub-pathway | Superpathway | Mean (SD)  Good responders | Mean (SD)  Poor responders | FDR |
| --- | --- | --- | --- | --- | --- |
| 1,5-anhydroglucitol (1,5-AG) | Glycolysis, Gluconeogenesis, and Pyruvate Metabolism | Carbohydrate | 0.86 (0.36) | 0.22 (0.23) | 2.76×10^-17^ |
| mannose | Fructose, Mannose and Galactose Metabolism | Carbohydrate | 1.18 (0.38) | 2.03 (0.72) | 5.90×10^-12^ |
| glucose | Glycolysis, Gluconeogenesis, and Pyruvate Metabolism | Carbohydrate | 1.22 (0.37) | 2.05 (0.79) | 5.80×10^-10^ |
| fructose | Fructose, Mannose and Galactose Metabolism | Carbohydrate | 1.2 (0.37) | 1.79 (0.63) | 1.31×10^-6^ |
| pyroglutamine* | Glutamate Metabolism | Amino Acid | 1.24 (0.77) | 0.66 (0.48) | 4.65×10^-5^ |
| methyl glucopyranoside (alpha + beta) | Food Component/Plant | Xenobiotics | 2.57 (4.88) | 0.81 (0.74) | 8.59×10^-5^ |
| mannonate* | Food Component/Plant | Xenobiotics | 1.27 (0.4) | 1.89 (0.67) | 8.97×10^-5^ |
| 6-bromotryptophan | Tryptophan Metabolism | Amino Acid | 1.06 (0.33) | 0.8 (0.27) | 0.000517 |
| palmitoylcholine | Fatty Acid Metabolism (Acyl Choline) | Lipid | 1.3 (0.78) | 0.88 (0.59) | 0.000531 |
| linoleoylcholine* | Fatty Acid Metabolism (Acyl Choline) | Lipid | 1.25 (0.72) | 0.82 (0.52) | 0.000594 |
| gluconate | Food Component/Plant | Xenobiotics | 1.29 (0.4) | 1.87 (0.75) | 0.001394 |
| methylsuccinoylcarnitine | Leucine, Isoleucine and Valine Metabolism | Amino Acid | 1.31 (0.72) | 1.85 (0.93) | 0.001995 |
| 3-methoxytyrosine | Tyrosine Metabolism | Amino Acid | 1.01 (0.26) | 0.82 (0.26) | 0.002158 |
| ethyl beta-glucopyranoside | Food Component/Plant | Xenobiotics | 4.48 (7.51) | 1.85 (3.36) | 0.002256 |
| methionine | Methionine, Cysteine, SAM and Taurine Metabolism | Amino Acid | 1.02 (0.18) | 0.9 (0.14) | 0.002829 |
| choline | Phospholipid Metabolism | Lipid | 1.06 (0.21) | 0.95 (0.19) | 0.002829 |
| gamma-glutamylglutamine | Gamma-glutamyl Amino Acid | Peptide | 1.03 (0.29) | 0.81 (0.29) | 0.002829 |
| 3-methyl-2-oxobutyrate | Leucine, Isoleucine and Valine Metabolism | Amino Acid | 1.06 (0.27) | 1.27 (0.31) | 0.002829 |
| arachidonoylcholine | Fatty Acid Metabolism (Acyl Choline) | Lipid | 1.27 (0.75) | 0.87 (0.5) | 0.002829 |
| glycerol 3-phosphate | Glycerolipid Metabolism | Lipid | 1.13 (0.56) | 0.87 (0.43) | 0.006382 |
| 2-hydroxybutyrate/2-hydroxyisobutyrate | Glutathione Metabolism | Amino Acid | 1.14 (0.46) | 1.97 (1.62) | 0.00649 |
| N-methylproline | Urea cycle; Arginine and Proline Metabolism | Amino Acid | 2.41 (2.63) | 1.33 (2.02) | 0.010862 |
| fructosyllysine | Lysine Metabolism | Amino Acid | 1.18 (0.43) | 1.52 (0.57) | 0.010862 |
| gamma-glutamyltyrosine | Gamma-glutamyl Amino Acid | Peptide | 1.12 (0.36) | 0.92 (0.3) | 0.025442 |
| androsterone glucuronide | Androgenic Steroids | Lipid | 1.42 (1.45) | 0.84 (0.78) | 0.026499 |
| alpha-hydroxyisovalerate | Leucine, Isoleucine and Valine Metabolism | Amino Acid | 1.02 (0.49) | 1.68 (2.06) | 0.039332 |
| 11beta-hydroxyandrosterone glucuronide | Androgenic Steroids | Lipid | 1.45 (0.82) | 1.17 (0.85) | 0.039332 |
| 2-hydroxy-3-methylvalerate | Leucine, Isoleucine and Valine Metabolism | Amino Acid | 1.05 (0.47) | 1.57 (1.58) | 0.039538 |
| gamma-glutamylmethionine | Gamma-glutamyl Amino Acid | Peptide | 1.1 (0.34) | 0.91 (0.34) | 0.046044 |
| cortisone | Corticosteroids | Lipid | 0.98 (0.4) | 1.13 (0.43) | 0.047957 |

**Table S2A**. Top metabolites differentiating good responders from poor responders in females.

| Metabolites | Sub-pathway | Superpathway | Estimate | SE | p-value | FDR |
| --- | --- | --- | --- | --- | --- | --- |
| glucose | Glycolysis, Gluconeogenesis, and Pyruvate Metabolism | Carbohydrate | -1.69104 | 0.236973 | 1.47×10^-9^ | 1.03×10^-6^ |
| 1,5-anhydroglucitol (1,5-AG) | Glycolysis, Gluconeogenesis, and Pyruvate Metabolism | Carbohydrate | 1.39404 | 0.19911 | 2.49×10^-9^ | 1.03×10^-6^ |
| mannose | Fructose, Mannose and Galactose Metabolism | Carbohydrate | -1.36056 | 0.220601 | 6.81×10^-8^ | 1.87×10^-5^ |
| fructose | Fructose, Mannose and Galactose Metabolism | Carbohydrate | -1.22175 | 0.227312 | 1.32×10^-6^ | 0.000272 |
| pyruvate | Glycolysis, Gluconeogenesis, and Pyruvate Metabolism | Carbohydrate | -1.25863 | 0.246252 | 3.52×10^-6^ | 0.00058 |
| mannonate* | Food Component/Plant | Xenobiotics | -1.06055 | 0.218088 | 8.71×10^-6^ | 0.001197 |
| 3-methyl-2-oxobutyrate | Leucine, Isoleucine and Valine Metabolism | Amino Acid | -1.11719 | 0.241954 | 2.10×10^-5^ | 0.002476 |
| 2-hydroxybutyrate/2-hydroxyisobutyrate | Glutathione Metabolism | Amino Acid | -1.09703 | 0.257785 | 7.43×10^-5^ | 0.007662 |
| choline | Phospholipid Metabolism | Lipid | 0.982533 | 0.2479 | 0.000199 | 0.018253 |
| alpha-hydroxyisocaproate | Leucine, Isoleucine and Valine Metabolism | Amino Acid | -0.93167 | 0.241271 | 0.000279 | 0.020772 |
| cortisone | Corticosteroids | Lipid | -1.14116 | 0.294874 | 0.000282 | 0.020772 |
| ethyl beta-glucopyranoside | Food Component/Plant | Xenobiotics | 1.071353 | 0.278017 | 0.000302 | 0.020772 |
| erythronate* | Aminosugar Metabolism | Carbohydrate | -0.98109 | 0.261161 | 0.000392 | 0.024862 |
| alpha-hydroxyisovalerate | Leucine, Isoleucine and Valine Metabolism | Amino Acid | -0.89256 | 0.24 | 0.000442 | 0.026064 |
| methyl glucopyranoside (alpha + beta) | Food Component/Plant | Xenobiotics | 1.302198 | 0.344564 | 0.00049 | 0.026965 |
| epiandrosterone sulfate | Androgenic Steroids | Lipid | 0.886771 | 0.248937 | 0.000735 | 0.037906 |
| glycerol | Glycerolipid Metabolism | Lipid | -0.67661 | 0.195738 | 0.001011 | 0.047902 |
| gluconate | Food Component/Plant | Xenobiotics | -0.80145 | 0.233339 | 0.001082 | 0.047902 |
| 3-methyl-2-oxovalerate | Leucine, Isoleucine and Valine Metabolism | Amino Acid | -0.85335 | 0.249244 | 0.001119 | 0.047902 |
| gamma-glutamylglutamine | Gamma-glutamyl Amino Acid | Peptide | 0.886613 | 0.259878 | 0.001161 | 0.047902 |
| fructosyllysine | Lysine Metabolism | Amino Acid | -0.90173 | 0.268756 | 0.00138 | 0.049546 |
| glycerol 3-phosphate | Glycerolipid Metabolism | Lipid | 0.900731 | 0.269225 | 0.00142 | 0.049546 |
| pyroglutamine* | Glutamate Metabolism | Amino Acid | 0.79946 | 0.239673 | 0.001464 | 0.049546 |
| 1-arachidonoyl-GPA (20:4) | Lysophospholipid | Lipid | -1.07869 | 0.309034 | 0.00147 | 0.049546 |
| 1-palmitoyl-2-palmitoleoyl-GPC (16:0/16:1)* | Phosphatidylcholine (PC) | Lipid | -0.88848 | 0.267027 | 0.001501 | 0.049546 |

**Table S2B**. Top metabolites differentiating good responders from poor responders in males.

| Metabolites | Sub-pathway | Superpathway | Estimate | SE | p-value | FDR |
| --- | --- | --- | --- | --- | --- | --- |
| 1,5-anhydroglucitol (1,5-AG) | Glycolysis, Gluconeogenesis, and Pyruvate Metabolism | Carbohydrate | 1.434606 | 0.177096 | 1.75×10^-11^ | 1.45×10^-8^ |
| mannose | Fructose, Mannose and Galactose Metabolism | Carbohydrate | -1.10659 | 0.214053 | 2.36×10^-6^ | 0.000977 |
| pyroglutamine* | Glutamate Metabolism | Amino Acid | 0.937469 | 0.199736 | 1.37×10^-5^ | 0.003781 |
| 6-bromotryptophan | Tryptophan Metabolism | Amino Acid | 1.030816 | 0.235932 | 4.44×10^-5^ | 0.009176 |
| fructose | Fructose, Mannose and Galactose Metabolism | Carbohydrate | -1.06018 | 0.247896 | 6.16×10^-5^ | 0.010182 |
| glucose | Glycolysis, Gluconeogenesis, and Pyruvate Metabolism | Carbohydrate | -0.93536 | 0.221749 | 7.56×10^-5^ | 0.010424 |
| sphingomyelin (d18:2/18:1)* | Sphingomyelins | Lipid | 0.902101 | 0.222938 | 0.000137 | 0.015973 |
| 1-palmitoyl-GPA (16:0) | Lysophospholipid | Lipid | 1.098247 | 0.266342 | 0.000155 | 0.015973 |
| methyl glucopyranoside (alpha + beta) | Food Component/Plant | Xenobiotics | 1.207116 | 0.317196 | 0.000368 | 0.033857 |
